# Detecting hypoxia *in vitro* using ^18^F-pretargeted IEDDA “click” chemistry in live cells†

**DOI:** 10.1039/d1ra02482e

**Published:** 2021-06-07

**Authors:** Louis Allott, Cen Chen, Marta Braga, Sau Fung Jacob Leung, Ning Wang, Chris Barnes, Diana Brickute, Laurence Carroll, Eric O. Aboagye

**Affiliations:** Comprehensive Cancer Imaging Centre, Faculty of Medicine, Department of Surgery and Cancer, Imperial College London, Hammersmith Hospital Du Cane Road London W12 0NN UK eric.aboagye@imperial.ac.uk; Positron Emission Tomography Research Centre, Faculty of Health Sciences, University of Hull Cottingham Road Kingston upon Hull HU6 7RX UK; Russell H. Morgan Department of Radiology and Radiological Sciences, Johns Hopkins Medical Institutions Baltimore Maryland USA

## Abstract

We have exemplified a pretargeted approach to interrogate hypoxia in live cells using radioactive bioorthogonal inverse electron demand Diels–Alder (IEDDA) “click” chemistry. Our novel ^18^F-tetrazine probe ([^18^F]FB-Tz) and 2-nitroimidazole-based TCO targeting molecule (**8**) showed statistically significant (*P* < 0.0001) uptake in hypoxic cells (*ca.* 90 %ID per mg) *vs.* normoxic cells (<10 %ID per mg) in a 60 min incubation of [^18^F]FB-Tz. This is the first time that an intracellularly targeted small-molecule for IEDDA “click” has been used in conjunction with a radioactive reporter molecule in live cells and may be a useful tool with far-reaching applicability for a variety of applications.

## Abstract 2

Bioorthogonal IEDDA “click” can interrogate intracellular hypoxia using a radioactive reporter molecule.

## Graphical abstract

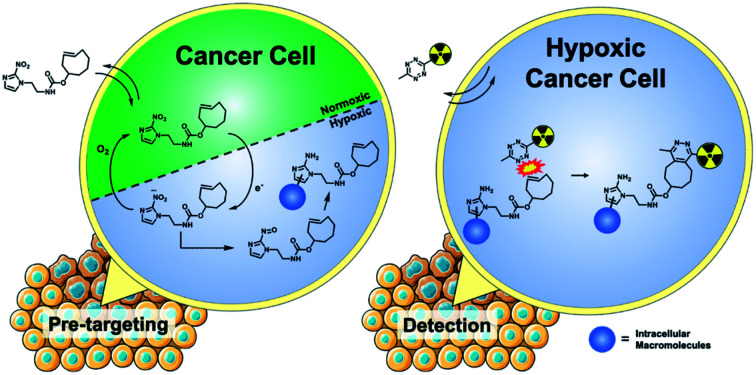

## Introduction

An ever-growing toolbox of “click” chemistries offers rapid, chemoselective ligation in aqueous reaction milieu.^1^ The inverse electron demand Diels–Alder (IEDDA) “click” reaction between tetrazine (Tz) and *trans*-cyclooctene (TCO) under physiological conditions (10^4^–10^5^ M^−1^ s^−1^) has been heavily investigated by the research community, particularly for performing bioorthogonal reactions.^1–5^ The commercial availability of Tz and TCO intermediates has facilitated the rapid and convenient assembly of complex conjugates and has conveniently placed biorthogonal chemistry in the hands of scientists. This technology has been used to visualise and quantify intracellular and extracellular targets by live cell fluorescence imaging and the medical imaging community has developed a new field of pretargeted positron emission tomography (PET) imaging for the *in vivo* detection of TCO-modified monoclonal antibodies (mAb).^6–11^ The study of *in vitro* intracellular targets in live cells has predominantly focused on fluorescence imaging, where a functionalised biomolecule bearing a tetrazine-reactive handle (*e.g.* TCO) accumulates within a cell which bioorthogonally reacts with a fluorescent-Tz reporter molecule;^12,13^ surprisingly, the use of radioactive reporter molecules in live cells has not been reported. Detecting radioactive species is a highly sensitive and quantitative technique, and radiolabelled reporter molecules may overcome the challenges associated with quantifying a fluorescence signal by microscopy and negate the physical limitations of some fluorophores, such as photobleaching.^14^

**Fig. 1:**
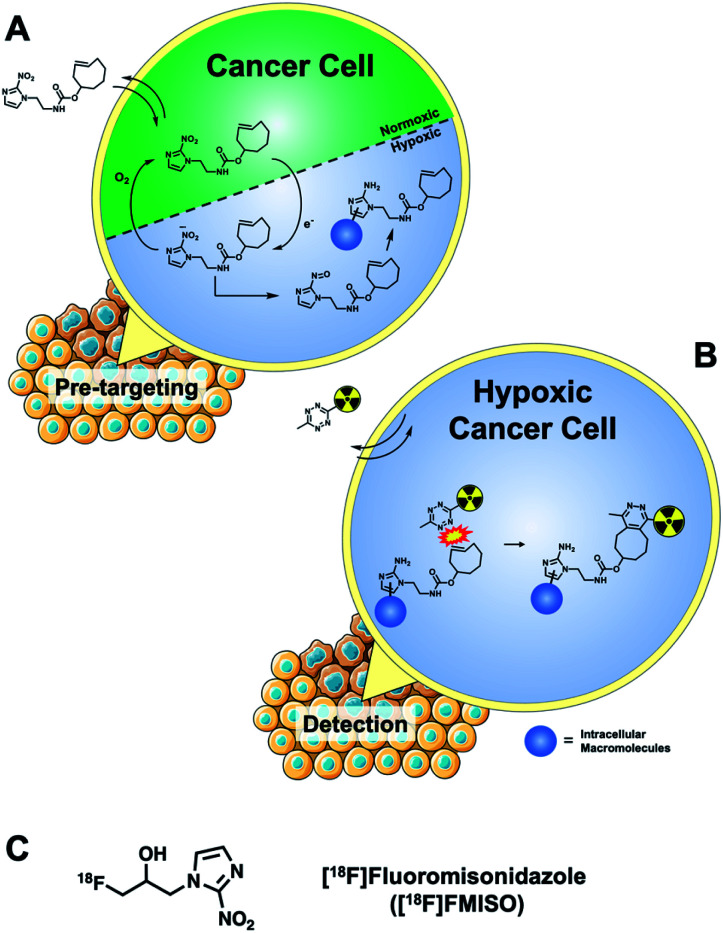
(A) Mechanism of 2-nitroimidazole retention in hypoxic cells exemplified by a TCO-containing derivative for intracellular pretargeted detection; (B) detecting the accumulation and retention of the TCO-bearing targeting molecule in hypoxic cells using a cell permeable radiolabelled tetrazine. (C) Structure of a fluorine-18 radiolabelled hypoxia radiotracer ([^18^F]FMISO).

We report a pretargeted IEDDA “click” method for detecting hypoxia in cancer cells with a small molecule 2-nitroimidazole targeting vector and a cell-permeable ^18^F-radiolabelled tetrazine. The 2-nitroimidazole pharmacophore accumulates in hypoxic cells *via* a well-known redox mechanism (Fig. 1); firstly, the compound diffuses passively across the cell membrane and the nitro-group is enzymatically reduced to a reactive species inside the cell. Under normoxic conditions, the nitro species is re-oxidized and can diffuse out of the cell. Under hypoxic conditions, the nitro species is further reduced to a nitroso moiety and ultimately to a primary amine; the reactive intermediates bind covalently to intracellular macromolecules and are thereby trapped.^15^ Fluorine-18 PET radiotracers based around the 2-nitroimidazole pharmacophore have been developed (*i.e.* [^18^F]fluoromisonidazole, [^18^F]FMISO, Fig. 1C) but their cellular uptake is low, owed to the difference between the physical half-life of the isotope and the time required for sufficient retention by the aforementioned redox mechanism (*ca.* 3–4 h).^16–20^ By using a pretargeted approach, we hypothesised that a 2-nitroimidazole pharmacophore bearing a TCO group, when allowed to accumulate inside the cell over a longer period of time (12 h) with follow-up incubation of an ^18^F-tetrazine (60 min incubation), may give better sensitivity of detection and higher *in vitro* accumulation than is possible with conventional radiotracers (Fig. 1).

## Results & discussion

**Scheme 1:**
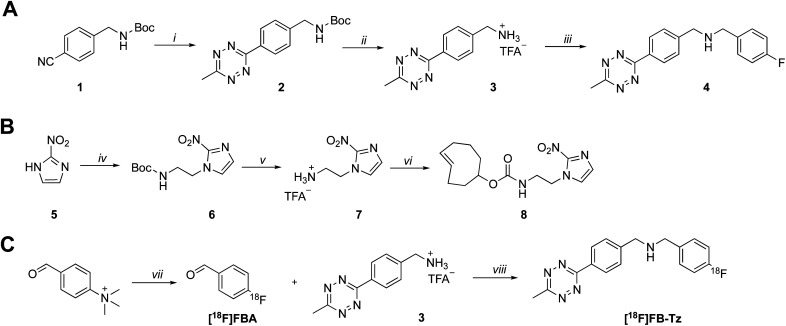
Synthesis of (A) tetrazine radiochemistry precursor (**3**) and reference standard (**4**) and (B) (*E*)-cyclooct-3-en-1-yl(2-(2-nitro-1*H*-imidazol-1-yl)ethyl)carbamate (**8**). Reaction conditions: (i) hydrazine hydrate, Ni(OTf)_2_, MeCN, 60 °C, 48 h; (ii) trifluoroacetic acid, DCM, RT, 2 h; (iii) 4-fluorobenzaldehyde, NaCNBH_3_, RT; (iv) 2-(Boc-amino)ethyl bromide, K_2_CO_3_, DMF, 60 °C, 16 h; (v) trifluoroacetic acid, DCM, RT, 2 h; (vi) TCO-NHS ester, triethylamine, DMF, RT, 16 h. (C) Radiosynthesis of [^18^F]FB-Tz; reaction conditions: (vii) [^18^F]KF, KHCO_3_, K_222_, MeCN, 90 °C, 10 min; (viii) MeCN, 50 °C, 20 min followed by flowing the reaction mixture through a custom built BH_3_CN^−^ cartridge.^21^

A simple TCO-modified 2-nitroimidazole was required for the *in vitro* pretargeted detection of hypoxia. (*E*)-Cyclooct-3-en-1-yl(2-(2-nitro-1*H*-imidazol-1-yl)ethyl)carbamate (**8**) was synthesised in three steps and comprised of the essential 2-nitroimidazole pharmacophore, a short alkyl linker and a TCO moiety for bioorthogonal IEDDA “click’ chemistry (Scheme 1B). Firstly, 2-nitroimidazole (**5**) was reacted with 2-(boc-amino)ethyl bromide to install the short alkyl chain linker (**6**). The boc-protecting group was hydrolysed under acidic conditions, producing a primary alkylammonium salt (**7**) which was quenched under basic conditions followed by the addition of TCO-NHS ester to produce compound **8** in a 53% yield.

**Fig. 2:**
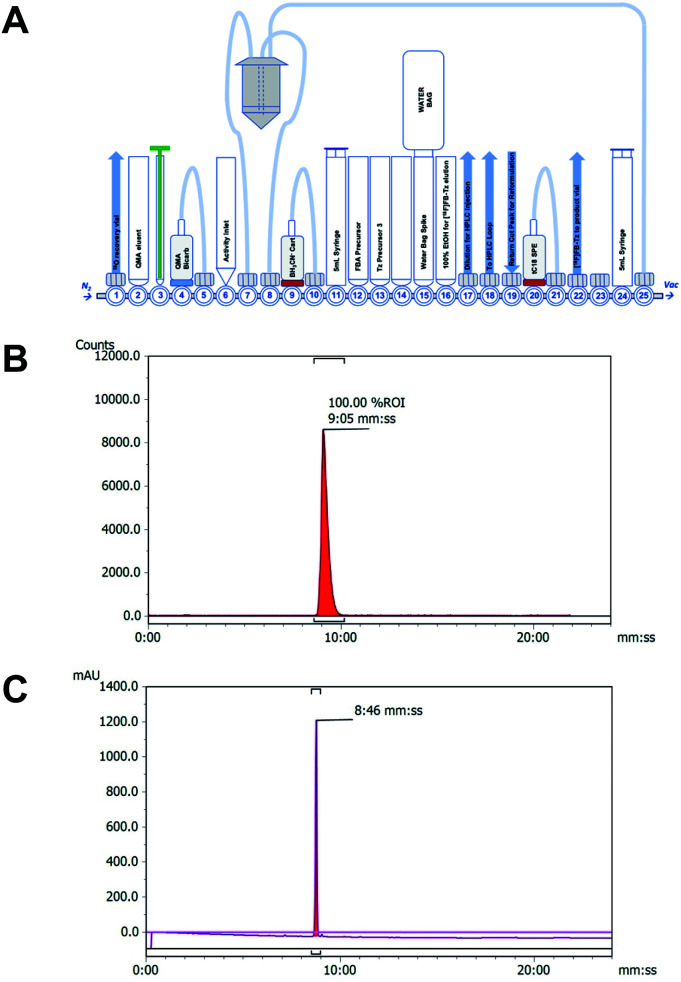
(A) Schematic representation of the FASTLab™ cassette for the automated radiosynthesis and purification of [^18^F]FB-Tz; (B) representative radio-HPLC chromatograms showing purified [^18^F]FB-Tz (*t*_R_ = 9:05 mm:ss); (C) representative UV-HPLC chromatogram showing an authentic reference standard (*t*_R_ = 8:46 mm:ss). There is a 20 s delay between the inline UV-detector and γ-detector on the analytical HPLC system used to run these samples.

A cell-permeable ^18^F-tetrazine ([^18^F]FB-Tz) was synthesised which was required to passively diffuse across the cell membrane of all cells without discrimination, with selective retention of [^18^F]FB-Tz in cells containing the retained TCO moiety (Scheme 1); this predicates a lipophilic tetrazine (log *D*_7.5_ > 1). Using simple reductive amination radiochemistry and the 4-[^18^F]fluorobenzaldehyde ([^18^F]FBA) prosthetic group with a previously reported automated radiolabelling methodology developed in our group on the GE FASTLab™ platform (Fig. 2A), we synthesised [^18^F]FB-Tz from precursor **3** (Scheme 1C). [^18^F]FB-Tz was produced in a 8.7 ± 1.1 non-decay corrected radiochemical yield (RCY n.d.c), a molar activity of 5 GBq μmol^−1^ and >96% radiochemical purity in 80 min (Fig. 2C and D).^21^ [^18^F]FB-Tz was lipophilic with an experimental log *D*_7.5_ of 1.14 ± 0.03.

With both the targeting molecule (**8**) and the radioactive reporter molecule ([^18^F]FB-Tz) in hand, the approach was evaluated biologically in hypoxic and normoxic cancer cell lines.

### Biological evaluation in live cells

Compound [^18^F]FB-Tz and compound **8** were evaluated *in vitro* using EMT6 and HCT116 cancer cell lines. Cells were treated with compound **8** at a range of concentrations (0.01–10 μM) and cultured for 24 h either under normoxia or hypoxia (Oxoid Anaerogen chamber; 0.1% O_2_). [^18^F]FB-Tz was then incubated with the pre-treated cells for 60 min under normal culture conditions, followed by rigorous washing of the cells with ice cold PBS before γ-counting.

**Fig. 3:**
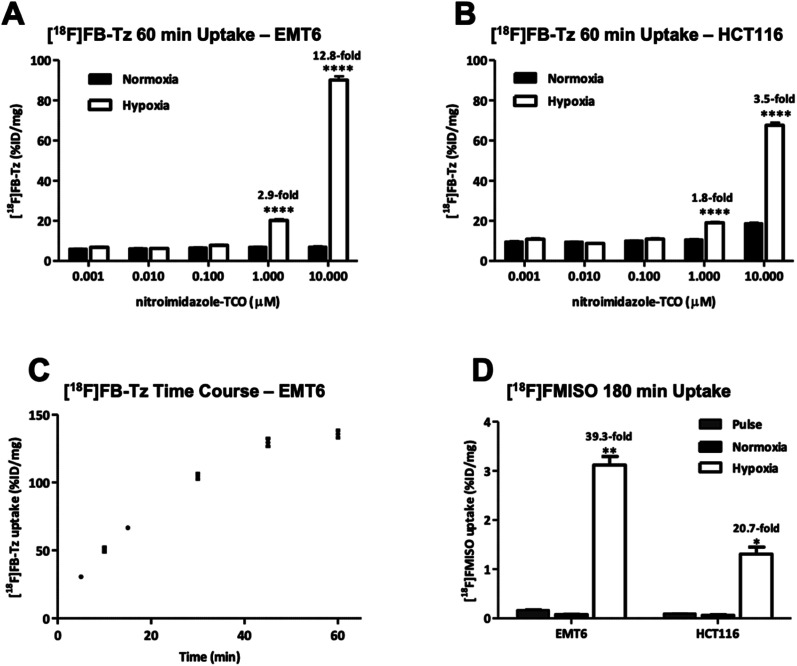
The *in vitro* uptake of [^18^F]FB-Tz in (A) EMT6 and (B) HCT116 cells. Cells were treated with **8** (0.01–10 μM) and cultured under normoxia or hypoxia (0.1% O_2_) conditions for 24 h prior to radioactive uptake experiment: 0.74 MBq per well of [^18^F]FB-Tz was added to cells and incubated for 60 min. (C) Time course of [^18^F]FB-Tz uptake in EMT6 cells *in vitro*. Cells were treated with 8 (10 μM) for 24 h under hypoxia before incubation with 0.74 MBq per well of [^18^F]FB-Tz for 5, 10, 15, 30, 45 and 60 min. (D) Radioactive uptake of [^18^F]FMISO (1.48 MBq per well) for 0 min (pulse) or 3 h under normoxia or hypoxia conditions in EMT6 and HCT116. Data were expressed as a percentage of total radioactivity incorporated into cells, normalized for total cellular protein. Mean values ± SD (*n* = 6; *P < 0.05, **P < 0.01 and ****P < 0.0001).

The *in vitro* uptake (Fig. 3A and B) showed a concentration-dependent increase in radioactivity accumulation in hypoxic cells for both EMT6 and HCT116 cell lines with increasing concentrations of **8** (1–10 μM). While incubation of **8** at concentrations between 0.001 and 0.1 μM had little impact on radioactive accumulation in both cell lines, incubation with 1 or 10 μM led to remarkable uptake; reaching 90.2 ± 4.2 %ID per mg in EMT6 cells and 67.7 ± 2.9 %ID per mg in HCT116 cells at 10 μM; notably, this was specific to hypoxia, with cells exhibiting a statistically significant (*P* < 0.0001) increase in radioactive accumulation under hypoxia (12.8-fold in EMT6 and 3.5-fold in HCT116) compared to normoxia conditions, which remained consistently low (<10 %ID per mg) at all concentrations of **8** studied. Radioactive accumulation is likely to be dependent upon the influence of the intracellular concentration of **8** on IEDDA reaction efficiency, and the molar activity of [^18^F]FB-Tz; therefore, radioactive uptake in the lower concentration range of **8** (0.001–0.1 μM) may improve with high molar activity ^18^F-tetrazines by reducing competition with non-radioactive ^19^F-tetrazine equivalent for the limited availability reactive TCO inside the cell. This was not further explored in this study. The *in vitro* uptake kinetics of [^18^F]FB-Tz in EMT6 cells incubated with 10 μM of **8** was determined; between 1 and 2-fold increase in radioactive uptake was observed every 5 min at the early time points (5–30 min), which plateaued at 45–60 min, suggesting rapid *in vitro* kinetics (Fig. 1C). The toxicity of **8** was evaluated *in vitro* using HCT116 and HepG2 cells to determine if pre-incubation at high concentrations perturbs the biological system under study. Incubating **8** in concentrations up to 100 μM showed no significant membrane damage as determined by LDH activity. The cytotoxicity of **8** incubated in concentrations up to 100 μM did did not inhibit/minimally inhibited cell growth in the cell lines over the 72 hour treatment used in the study. Taken together, compound **8** exhibited low toxicity at high concentrations (ESI, Fig. S10 and S11†)

### Comparison to a hypoxia radiotracer ([^18^F]FMISO)

The uptake of the PET radiopharmaceutical [^18^F]FMISO was used as a “gold standard” to demonstrate the detection of cellular hypoxia *via* radioactive uptake assay in live cells. [^18^F]FMISO was incubated in both hypoxic/normoxic EMT6 and HCT116 cell lines for 3 h to compare absolute and relative uptake with the pretargeted approach. The incubation time of 3 h was selected based on previous studies which showed optimal uptake at later time-points.^18,22–24^ The absolute uptake values of [^18^F]FMISO (3.1 ± 0.4 and 1.3 ± 0.3 %ID per mg for EMT6 and HCT116, respectively) were considerably lower compared to the pretargeted approach (90.2 ± 4.2 and 67.7 ± 2.9 %ID per mg in EMT6 and HCT116, respectively). The hypoxic : normoxic (H : N) ratio of [^18^F]FMISO was higher than for the pretargeted approach (39.3 *vs.* 12.8 in EMT6 and 20.7 *vs.* 3.5 in HCT116 cells) due to a degree of non-specific uptake of the relatively lipophilic [^18^F]FB-Tz in normoxic cells (<10 %ID per mg protein). The uptake experiments confirmed that radioactive accumulation was specific to cellular hypoxia, however the specificity for detecting hypoxia was dependent upon higher concentrations (1–10 μM) of **8**.

## Conclusions

The bioorthogonal IEDDA “click” reaction between a radiolabelled cell-permeable reporter molecule ([^18^F]FB-Tz) and **8** in live cells was successful and significantly differentiated hypoxic and normoxic cells by a 12.8-fold increase in relative uptake. The use of [^18^F]FMISO confirmed relevant hypoxia levels in our model system, evidenced by cellular uptake of 3.1 ± 0.4 %ID per mg of within 180 min. By pretargeting cellular macromolecules for 24 h under similar hypoxic conditions, we were able to detect the consequent hypoxic adducts, evidenced by [^18^F]FMISO cellular uptake of 90.2 ± 4.2 %ID per mg within 60 min. Two key differences between the pre-targeting approach and the direct use of a radiolabelled nitroimidazole are noteworthy. First, even though the uptake of [^18^F]FB-Tz was rapid, the pretargeted approach leads to a degree of non-specific accumulation of TCO in normoxic cells and consequently [^18^F]FB-Tz normoxia labelling, leading to lower hypoxic : normoxic ratio compared to the [^18^F]FMISO approach. Second, pretargeting of cellular macromolecules combined with the use of [^18^F]FB-Tz was dependent on drug concentration, and only observed at 1–10 μM of **8**. The lack of toxicity of **8** in the LDH assay (ESI, Fig. S10†) and ability to achieve similar high concentrations of 2-nitroimidazoles for imaging by magnetic resonance spectroscopy support the pretargeting approach.^25^ Furthermore, the physicochemical properties of **8** could be further optimised to achieve higher hypoxia : normoxia ratios. Beyond the immediate application of this technology to hypoxia measurements, the higher level of cellular accumulation (*ca.* 90 %ID per mg *vs.* 3 %ID per mg) could open up possibilities for the effective delivery of therapeutic radioisotopes, or drug payloads and subsequent monitoring of IEDDA reaction efficiency with [^18^F]FB-Tz uptake acting as a pharmacodynamic marker; however, the non-specific uptake of cell-permeable tetrazines may be a potential challenge to overcome in the utility of this strategy. We studied this approach in the context of cellular hypoxia, but the general mechanism and [^18^F]FB-Tz could be adapted and applied to other biological targets where a differential retention of TCO can be established within a population of cells. The described approach could be an important platform to answering biological questions where a sensitive detection method is required.

## Experimental

### Materials and methods

All reagents and solvents were purchased from commercial sources and were used without further purification unless otherwise stated. HPLC grade acetonitrile (MeCN), dichloromethane (DCM), ethyl acetate (EtOAc), hexane, methanol (MeOH), triethylamine (TEA), potassium carbonate (K_2_CO_3_), potassium bicarbonate (KHCO_3_) and trifluoroacetic acid (TFA) were purchased from Sigma Aldrich (Gillingham, Dorset, UK). (*E*)-cyclooct-4-enyl 2,5-dioxo-1-pyrrolidinyl carbonate (TCO-NHS ester) was purchased from Jena Bioscience (Jena, Germany). ^1^H and ^13^C NMR spectra were obtained using a Bruker 400 MHz spectrometer operating at room temperature. Chemical shifts (*δ*) are reported in parts per million (ppm) and residual solvent peaks have been used as an internal reference. Peak multiplicities have been abbreviated as follows: s (singlet), d (doublet), dd (double-doublet), m (multiplet). NMR spectra were analysed using MestReNova v11 (Santiago de Compostela, Spain). Accurate mass spectra were obtained *via* the Imperial College Department of Chemistry Mass Spectrometry service. [^18^F]Fluoride was produced by a GE PETtrace cyclotron by 16 MeV irradiation of enriched [^18^O]H_2_O target, supplied by Alliance Medical Radiopharmacy Ltd (Warwick, UK). The automated radiosynthesis of [^18^F]FB-Tz and [^18^F]FMISO was performed using the GE FASTlab™ automated synthesis module (GE Healthcare Life Sciences, Amersham, UK). The radiochemistry precursor (NITTP) for [^18^F]FMISO was purchased from ABX GmbH (Radeberg, Germany). Solid phase extraction (SPE) cartridges were purchased from Waters (Elstree, Hertfordshire, UK) and used according to the manufacturers recommended guidelines. The 4-formyl-*N*,*N*,*N*-trimethylanilinium trifluoromethanesulfonate precursor ([^18^F]FBA precursor) was synthesised following a literature procedure.^26^ Radioactive product identity was determined by RP-HPLC using an Agilent 1200 series instrument connected to a flow-ram detector (Lablogic, Sheffield, UK). The system was equipped with a Phenomenex Gemini 5μ C18 110 Å (150 × 4.6 mm) column; the mobile phase was A: H_2_O (0.1% TFA) and B: MeCN. The gradient was: 0–1 min, 95% A 1–16 min, 5% A 16–17 min, 95% A 17–20 min, 95% A at 1 mL min^−1^. Elution profiles were analysed using Laura software (Lablogic, Sheffield, UK).

### Synthesis

The general synthesis for compounds used in this work are shown in Scheme 1. The synthesis of tetrazine 3 (radiochemistry precursor) and 2-(2-nitro-1*H*-imidazol-1-yl)ethan-1-amine (**7**) are reported elsewhere.^21,27^

#### Synthesis of *N*-(4-fluorobenzyl)-1-(4-(6-methyl-1,2,4,5-tetrazin-3-yl)phenyl)methan amine (**4**)

(4-(6-Methyl-1,2,4,5-tetrazin-3-yl)phenyl)methanaminium trifluoroacetate salt (200 mg, 0.66 mmol) and 4-fluorobenzalehyde (81 mg, 0.66 mmol) was added to dry MeCN followed by the addition of sodium triacetoxyborohydride (212 mg, 1.00 mmol). The reaction was stirred for 16 h at ambient temperature. Solvent was removed *in vacuo* and the residue purified by column chromatography (1 : 1 EtOAc/hexane, silica) to yield the product as a pink solid (71 mg, 35%). ^1^H-NMR (400 MHz, chloroform-d) *δ* 8.54–8.43 (m, 2H), 7.55–7.47 (m, 2H), 7.30–7.22 (m, 2H), 7.00–7.22 (m, 2H), 7.00–6.90 (m, 2H), 3.84 (s, 2H), 3.75 (s, 2H), 3.02 (s, 3H). ^13^C-NMR (400 MHz, chloroform-d) *δ* 21.1, 52.7, 115.4, 128.06, 128.9, 129.8, 130.6, 135.6, 145.1, 160.8, 163.2, 164.0, 167.2. ^19^F-NMR (376 MHz, chloroform-d) *δ* −115.77. ESI-MS (*m*/*z*): [M + H]^+^ calcd for C_17_H_17_N_5_F, 310.1486; found, 310.1464.

#### Synthesis of (*E*)-cyclooct-3-en-1-yl(2-(2-nitro-1*H*-imidazol-1-yl)ethyl)carbamate (**8**)

2-(2-Nitro-1*H*-imidazol-1-yl)ethan-1-amine (7, 35.1 mg, 0.224 mmol) was dissolved in DMF (2 mL). Triethylamine (31.2 μL, 0.224 mmol) and (*E*)-cyclooct-4-enyl 2,5-dioxo-1-pyrrolidinyl carbonate (TCO-NHS ester, 50 mg, 0.187 mmol) were added and stirred at r.t. for 16 h. The crude was evaporated and purified by column chromatography (100% EtOAc, silica) to yield the product as a yellow powder (30 mg, 52% yield). ^1^H-NMR (400 MHz, chloroform-d) *δ* 7.07–6.91 (m, 2H), 5.70–5.17 (m, 3H), 4.57–4.34 (m, 2H), 4.32–4.17 (m, 1H), 3.60–3.43 (m, 2H), 2.38–2.17 (m, 3H), 1.94–1.73 (m, 4H), 1.72–1.36 (m, 3H). ^13^C-NMR (101 MHz, chloroform-d) *δ*: 29.7, 31.0, 32.5, 34.2, 38.6, 41.0, 49.4, 81.4, 126.7, 128.5, 133.0, 142.2, 156.3. ESI-MS (*m*/*z*): [M + H]^+^ calcd for C_14_H_21_N_4_O_4_, 309.1563; found, 309.1567.

### Radiochemistry

[^18^F]FB-Tz was synthesised using the GE FASTLab™ automated radiosynthesis platform *via* reductive amination radiochemistry between precursor 3 and 4-[^18^F]fluorobenzaldehyde ([^18^F]FBA) (Scheme 1). We described the automated procedure elsewhere;^21^ in brief, aqueous [^18^F]fluoride was dried for the radiosynthesis of [^18^F]FBA from the 4-formyl-*N*,*N*,*N*-trimethylanilinium trifluoromethansulfonate precursor and used without further purification. Compound **3** was dissolved in acetonitrile by the addition of triethylamine to quench the TFA-salt, then added to the vessel containing [^18^F]FBA. The imine intermediate was formed at 65 °C for 15 min, before the reaction mixture was flowed through a cartridge containing solid-supported cyanoborohydride to reduce the imine to the secondary amine. [^18^F]FB-Tz was purified by semi-preparative HPLC (Shimadzu LC20-AT pump attached to a custom-built system, equipped with an Agilent Eclipse XDB-C18, 5μ (250 × 9.4 mm) column. The mobile phase was 20% EtOH/80% sodium phosphate (58 mM, pH 2.4) at a flow rate of 4 mL min^−1^) and the desired product was diluted in water (30 mL), trapped on a tC18 SPE cartridge, dried under a flow of nitrogen and eluted in EtOH for biological use. [^18^F]FB-Tz was characterised by radio-HPLC to determine its identity (by comparison to an authentic reference standard) and purity (Fig. 2B and C).

### Cell culture

EMT6 murine breast cancer cells were a kind gift from Prof. Sofia Pascu (University of Bath, UK) and were grown in Waymouth medium (Thermo Fisher Scientific); HCT116 human colorectal cancer cells (ATCC) were grown in Dulbecco Modified Eagle Medium (DMEM, Sigma-Aldrich). All media were supplemented with 1% l-glutamine and 2% penicillin–streptomycin (Life Technologies). Waymouth medium contained 15% fetal calf serum, with 10% fetal calf serum added to DMEM. All cells were cultured at 37 °C in a humidified atmosphere containing 5% CO_2_. All cells were routinely tested for mycoplasma and typically not passaged for longer than three months.

### *In vitro* uptake of [^18^F]FB-Tz

EMT6 and HCT116 cells were, respectively, plated at 8 × 10^4^ and 2.5 × 10^5^ cells per well in 6-well plates. After 24 h, cells were treated with **8** at indicated concentrations ranging from 0.01 to 10 μM and cultured under normoxia or within a hypoxia (Oxoid Anaerogen) chamber; 0.1% O_2_ for 24 h. Hypoxia was confirmed through the change of color of the indicator strip (Oxoid Resazurin, Thermo Fisher Scientific). On the day of the radioactive uptake experiment, fresh media containing 0.74 MBq of [^18^F]FB-Tz was added to individual wells (1 mL per well). Cells were incubated with the radiotracer for 60 min at 37 °C in a humidified condition of 5% CO_2_. Cells were then washed three times with ice-cold PBS and lysed in RIPA buffer (1 mL per well) for 10 min on ice. The radioactivity of 800 μL lysate from each sample was counted on a WIZARD2 Automatic Gamma Counter. Data were expressed as a percentage of incubated radioactive dose (ID), normalized to total cellular protein (%ID per mg) as measured by bicinchoninic acid (BCA) assay.

### *In vitro* uptake of [^18^F]FMISO

EMT6 and HCT116 cells were plated at 5 × 10^5^ cells per well in 6-well plates and allowed to settle for 24 h. On the day of the radioactive uptake experiment, fresh media containing 1.48 MBq of [^18^F]FMISO was added to individual wells (1 mL per well) and incubated for 0 min (pulse) or 3 h under normoxia or hypoxia conditions (Oxoid Anaerogen chamber, 0.1% O_2_) at 37 °C in a humidified condition of 5% CO_2_. Hypoxia was confirmed through the change of color of the indicator strip (Oxoid Resazurin, Thermo Fisher Scientific). Cells were then washed three times with ice-cold PBS and lysed in RIPA buffer (1 mL per well) for 10 min on ice. The radioactivity of 800 μL lysate from each sample was counted on a WIZARD2 Automatic Gamma Counter. Data were expressed as a %ID per mg.

### *In vitro* time course analysis of [^18^F]FB-Tz uptake

EMT6 cells were plated at 8 × 10^4^ cells per well in 6-well plates and allowed to attach for 24 h. Cells were then treated with **8** (10 μM) and incubated within a hypoxia chamber for 24 h. On the day of the radioactive uptake experiment, cells were incubated with fresh media containing 0.74 MBq per well of [^18^F]FB-Tz for 5, 10, 15, 30, 45 and 60 min at 37 °C in a humidified condition of 5% CO_2._ After incubation cells were washed, lysed and counted for radioactivity on a WIZARD2 Automatic Gamma Counter as described above. Data were expressed as %ID per mg.

### Lactate dehydrogenase (LDH) activity assay

Cell membrane integrity was assessed using a colorimetric LDH activity assay (Sigma Aldrich, Gillingham, UK). Experiments were performed in accordance to the manufacturer's instructions. In brief, HCT116 and HepG2 cell lines (5000 and 3000 cells per well, respectively) were incubated in 96-well plates for 4 h with compound **8**. Plates were equilibrated to ambient temperature before a sample (50 μL) was incubated with the Master Reaction Mix (50 μL) at 37 °C for 10 min before reading (450 nm). A positive control was included in the experiment, provided by the manufacturer. Background signal was subtracted from each data point and results were expressed as the quantity of LDH to catalyse the conversion of lactate into pyruvate to generate 1 μmol of NADH.

### Sulforhodamine B (SRB) assay

The *in vitro* toxicity of 8 was evaluated upon 72 h treatment in an SRB assay described elsewhere.^28^

### Statistical analysis

Data were analysed using GraphPad Prism v.8.2.1. The results were expressed as the mean values ± standard deviation (SD). The ANOVA was used for multiple comparisons and differences were defined as significant if *P* < 0.05.

## Conflicts of interest

There are no conflicts to declare.

## Supplementary Material

RA-011-D1RA02482E-s001
