# Supplementary material for: Detecting hypoxia in vitro using 18F-pretargeted IEDDA “click” chemistry in live cells
Source: RSC Adv. 2021 Jun 7;11(33):20335–41. doi: 10.1039/d1ra02482e (PMC8182949; doi:10.1039/d1ra02482e)
Supplement: RA-011-D1RA02482E-s001 [file RA-011-D1RA02482E-s001.pdf]

Electronic Supplementary Material (ESI) for RSC Advances.
This journal is © The Royal Society of Chemistry 2021

***RSC Advances***

# Detecting hypoxia *in vitro* using $^{18}$F-pretargeted IEDDA "click" chemistry in live cells

Louis Allott[a,b], Cen Chen[a], Marta Braga[a], Jacob S. F. Leung[a], Ning Wang[a], Chris Barnes[a], Diana Brickute[a], Laurence Carroll[a,c] and Eric O. Aboagye*[a]

[a] Comprehensive Cancer Imaging Centre, Imperial College London, Hammersmith Hospital, Du Cane Road, London, W12 0NN, UK. *Correspondence: eric.aboagye@imperial.ac.uk, ¥authors contributed equally.

[b] Positron Emission Tomography Research Centre, Faculty of Health Sciences, University of Hull, Cottingham Road, Kingston upon Hull, HU6 7RX, UK

[c] Russel H. Morgan Department of Radiology and Radiological Sciences, Johns Hopkins Medical Institutions, Baltimore, Maryland, USA

# Supporting Information

**Contents**

1.0 Materials and methods……………………………….……….…………………..Page 02
2.0 NMR Spectra…………………………………………………………..…………………Page 03
3.0 Mass Spectra of compound **8**……………………………………………..………..Page 06
4.0 Toxicity study………………………………………………………………………..……Page 07
5.0 References……………………………..………………………………..…………Page 08

## 1.0 Materials and Methods

All reagents and solvents were purchased from commercial sources and were used without further purification unless otherwise stated. HPLC grade acetonitrile (MeCN), dichloromethane (DCM), ethyl acetate (EtOAc), hexane, methanol (MeOH), triethylamine (TEA), potassium carbonate ($K_2CO_3$), potassium bicarbonate ($KHCO_3$) and trifluoroacetic acid (TFA) were purchased from Sigma Aldrich (Gillingham, Dorset, UK). (*E*)-cyclooct-4-enyl 2,5-dioxo-1-pyrrolidinyl carbonate (TCO-NHS ester) was purchased from Jena Bioscience (Jena, Germany). $^{1}H$ and $^{13}C$ NMR spectra were obtained using a Bruker 400 MHz spectrometer operating at room temperature. Chemical shifts (δ) are reported in parts per million (ppm) and residual solvent peaks have been used as an internal reference. Peak multiplicities have been abbreviated as follows: s (singlet), d (doublet), dd (double-doublet), m (multiplet). NMR spectra were analysed using MestReNova v11 (Santiago de Compostela, Spain). Accurate mass spectra were obtained *via* the Imperial College Department of Chemistry Mass Spectrometry service. [$^{18}F$]Fluoride was produced by a GE PETtrace cyclotron by 16 MeV irradiation of enriched [$^{18}O$]H2O target, supplied by Alliance Medical Radiopharmacy Ltd (Warwick, UK). The automated radiosynthesis of [$^{18}F$]FB-Tz and [$^{18}F$]FMISO was performed using the GE FASTlab™ automated synthesis module (GE Healthcare Life Sciences, Amersham, UK). The radiochemistry precursor (NITTP) for [$^{18}F$]FMISO was purchased from ABX Gmbh (Radeberg, Germany). Solid phase extraction (SPE) cartridges were purchased from Waters (Elstree, Hertfordshire, UK) and used according to the manufacturers recommended guidelines. The 4-formyl-*N,N,N*-trimethylanilinium trifluoromethanesulfonate precursor ([$^{18}F$]FBA precursor) was synthesised following a literature procedure [1]. Radioactive product identity was determined by RP-HPLC using an Agilent 1200 series instrument connected to a flow-ram detector (Lablogic, Sheffield, UK). The system was equipped with a Phenomenex Gemini 5μ C18 110 Å (150 × 4.6 mm) column; the mobile phase was A: $H_2O$ (0.1% TFA) and B: MeCN. The gradient was: 0 – 1 min, 95% A. 1 – 16 min, 5% A. 16 – 17 min, 95% A. 17 – 20 min, 95% A at 1 mL/min. Elution profiles were analysed using Laura software (Lablogic, Sheffield, UK). Semi-preparative RP-HPLC was performed using a Shimadzu LC20-AT pump attached to a custom-built system, equipped with an Agilent Eclipse XDB-C18, 5μ (250 x 9.4 mm) column. The mobile phase was 20% EtOH / 80% sodium phosphate (58 mM, pH 2.4) at a flow rate of 4 mL/min.

## 2.0 NMR Spectra

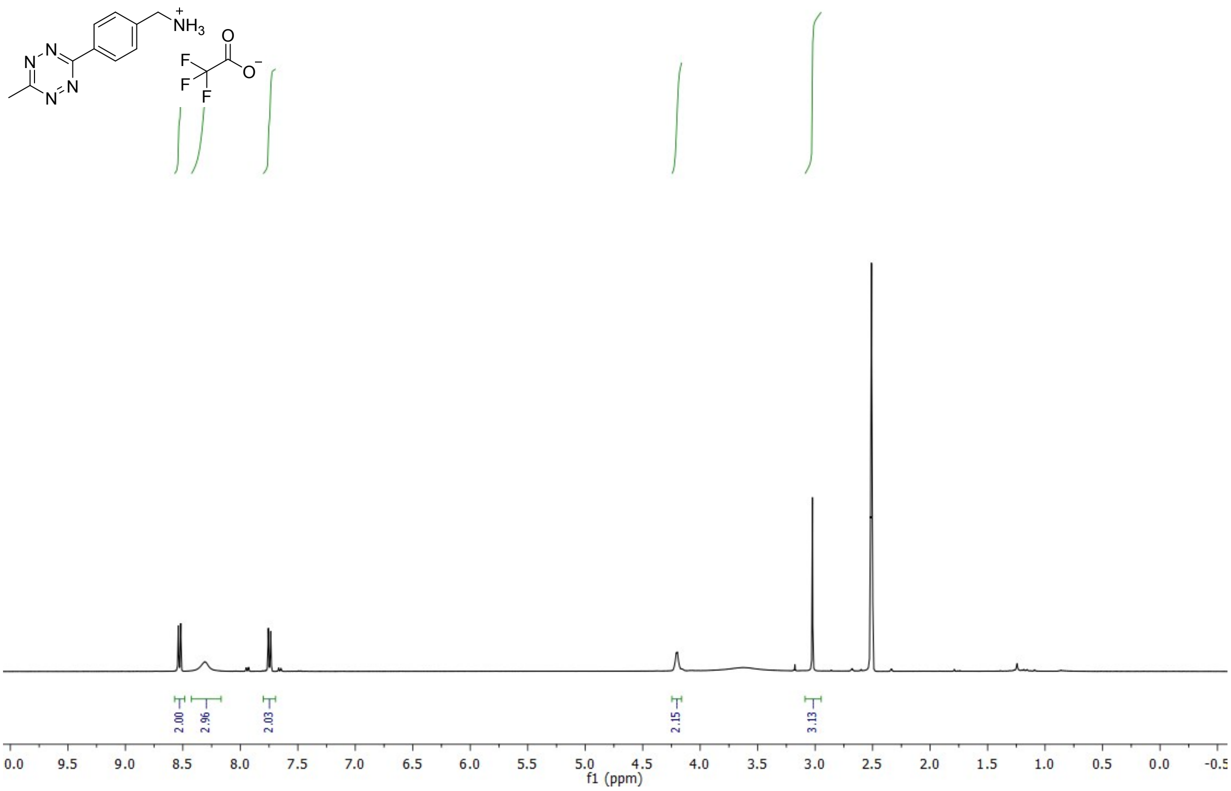


**Figure 1.** $^{1}$H-NMR of (4-(6-methyl-1,2,4,5-tetrazin-3-yl)phenyl)methanamine (**3**)

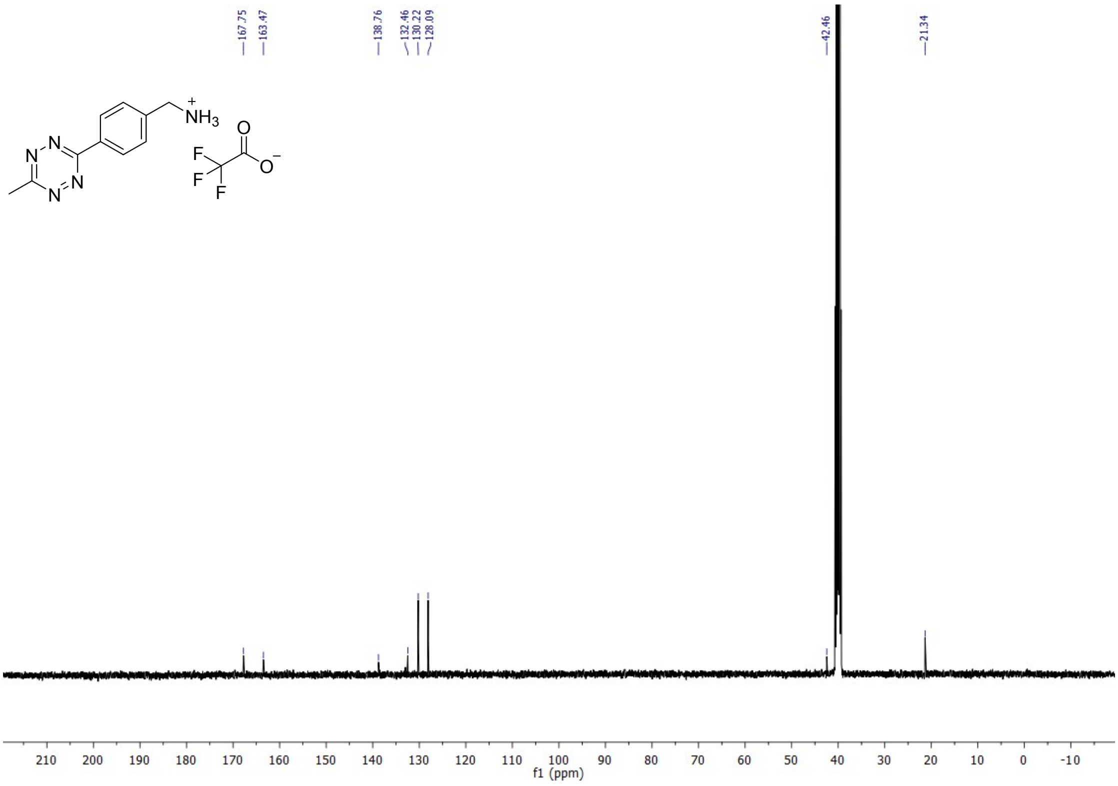


**Figure 2.** $^{13}$C-NMR of (4-(6-methyl-1,2,4,5-tetrazin-3-yl)phenyl)methanamine (**3**)

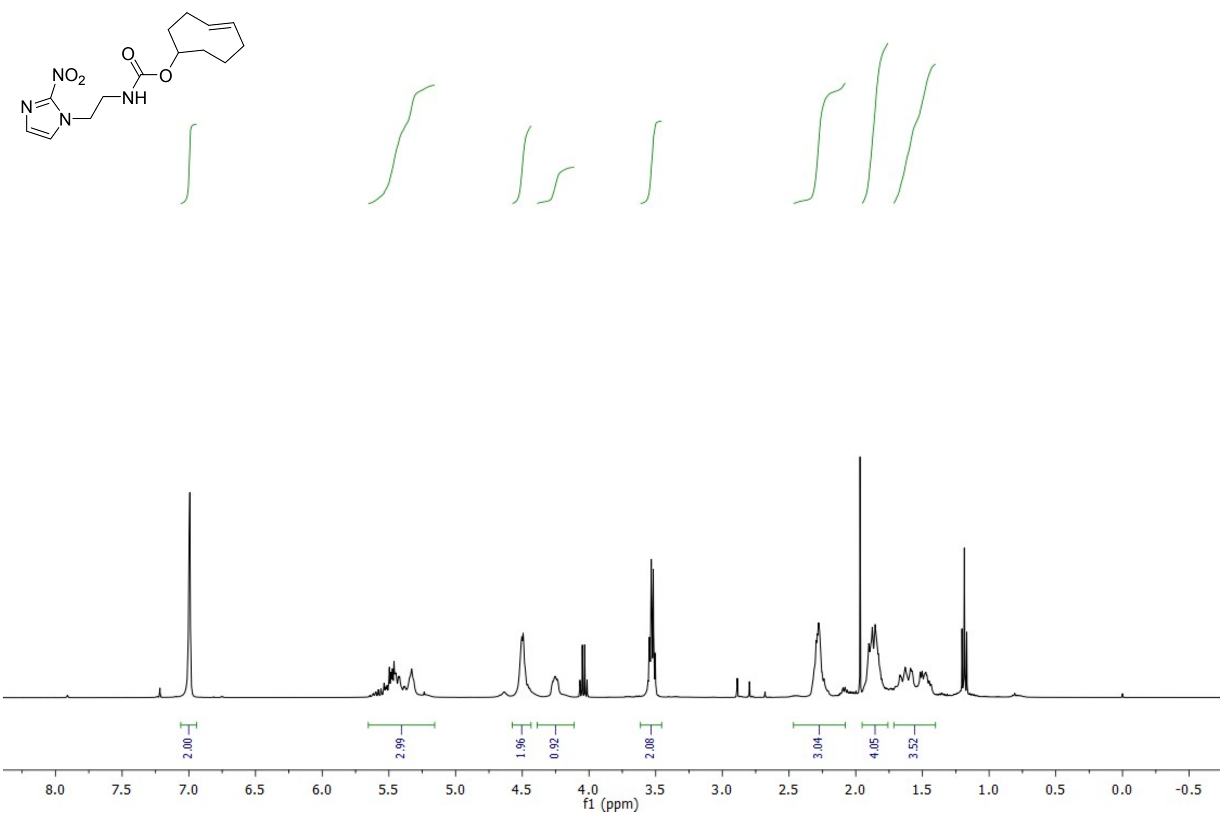


**Figure 3.** $^{1}$H-NMR of (*E*)-cyclooct-4-en-1-yl (2-(2-nitro-1H-imidazol-1-yl)ethyl) carbamate (**8**)

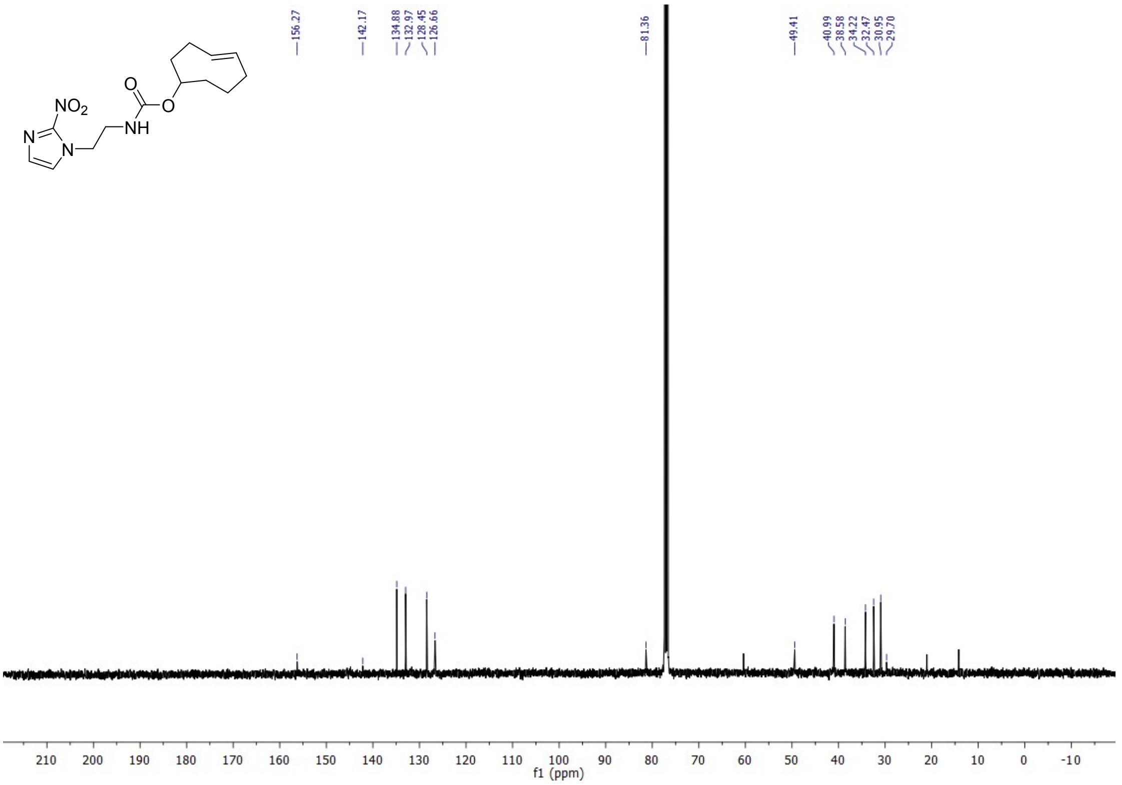


**Figure 4.** $^{13}$C-NMR of (*E*)-cyclooct-4-en-1-yl (2-(2-nitro-1H-imidazol-1-yl)ethyl) carbamate (**8**)

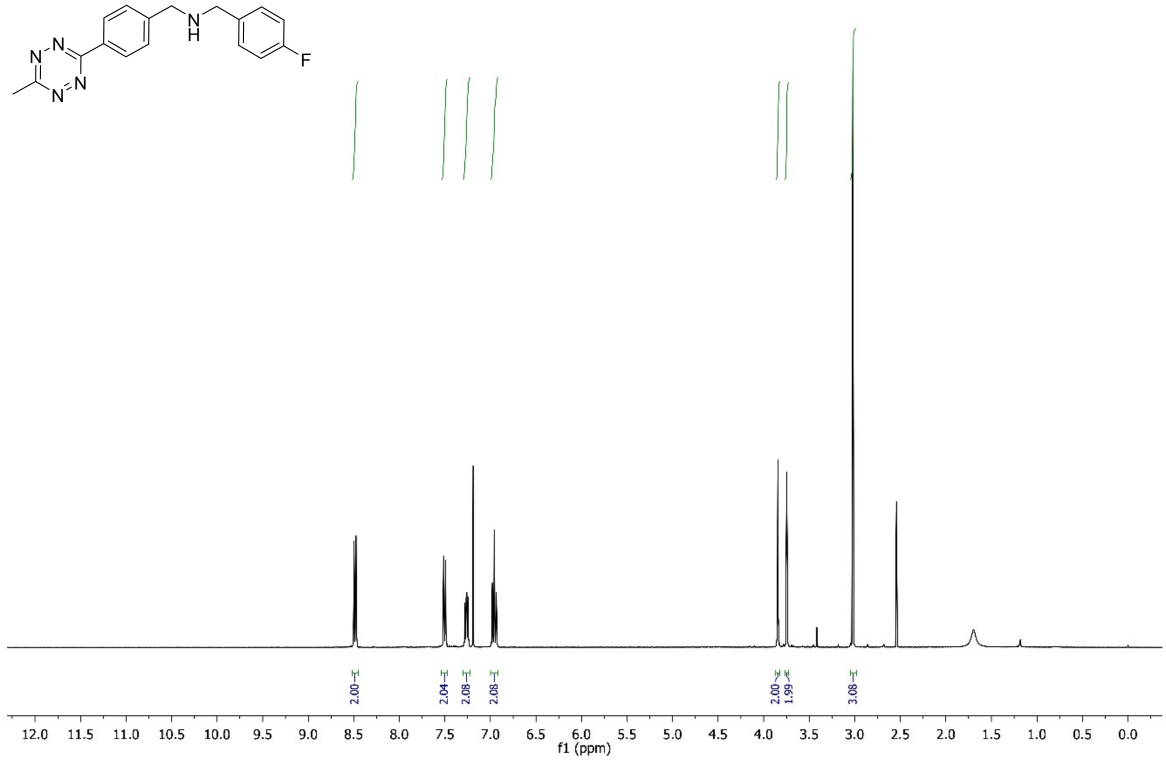


**Figure 5.** $^{1}$H-NMR of *N*-(4-fluorobenzyl)-1-(4-(6-methyl-1,2,4,5-tetrazin-3-yl)phenyl)methanamine (**4**)

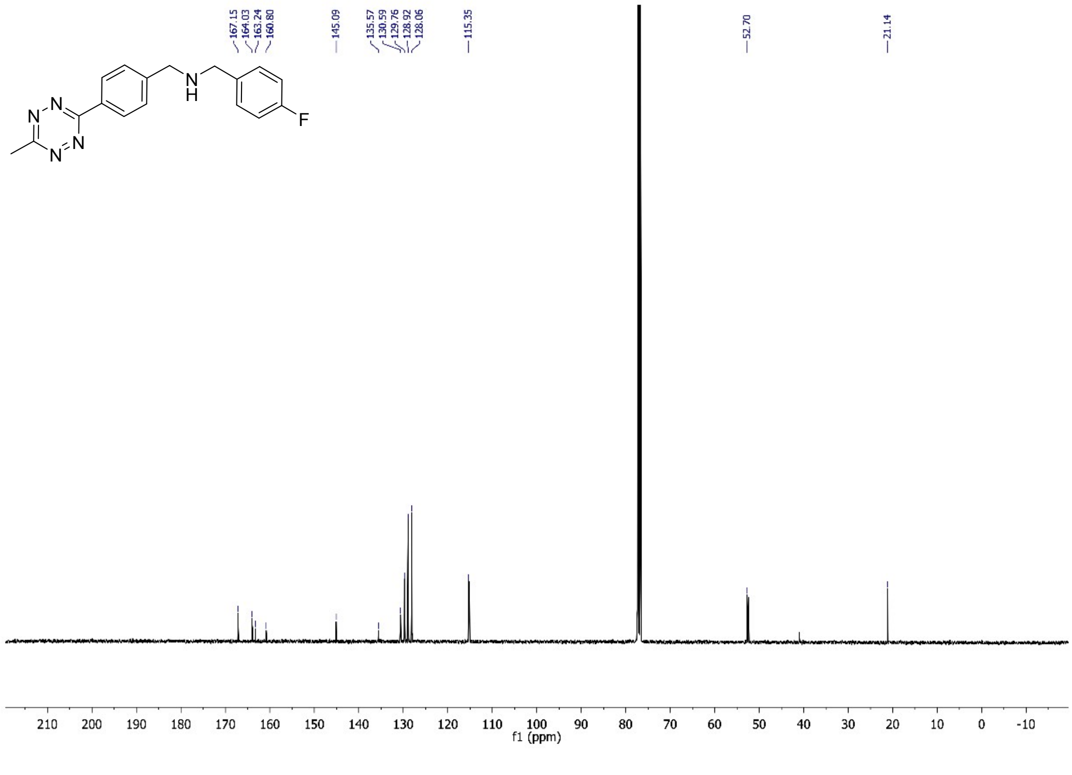


**Figure 6.** $^{13}$C-NMR of *N*-(4-fluorobenzyl)-1-(4-(6-methyl-1,2,4,5-tetrazin-3-yl)phenyl)methanamine (**4**)

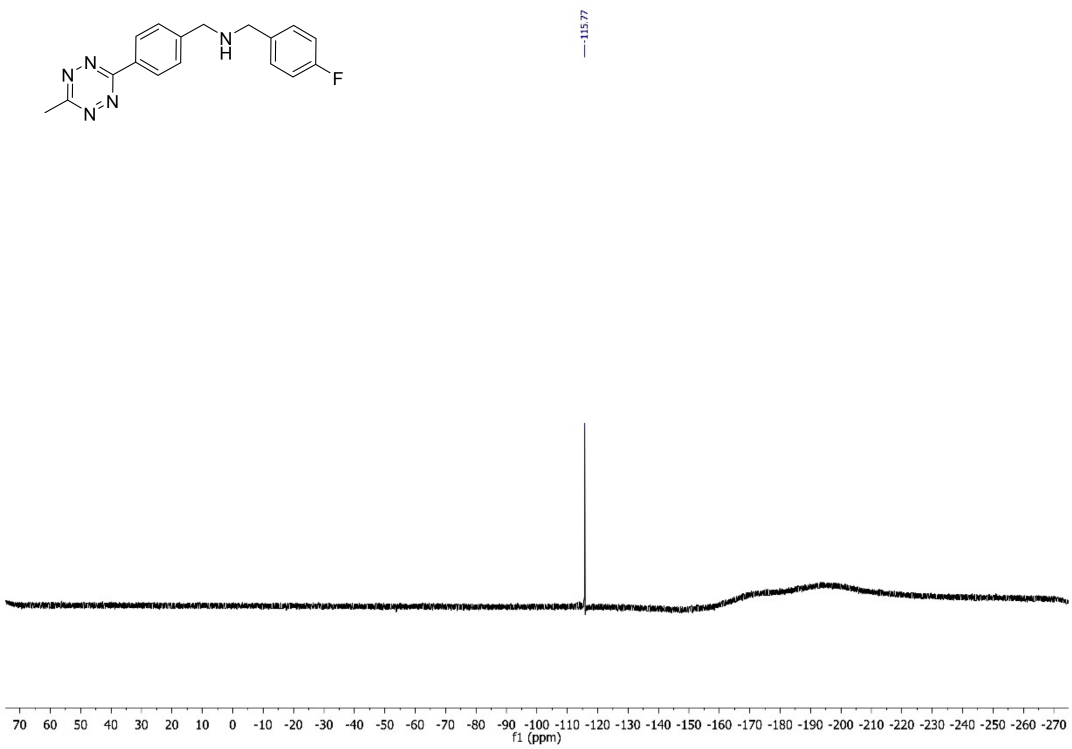


**Figure 7.** $^{19}$F-NMR of *N*-(4-fluorobenzyl)-1-(4-(6-methyl-1,2,4,5-tetrazin-3-yl)phenyl)methanamine (**4**)

### 3.0 Mass spectra

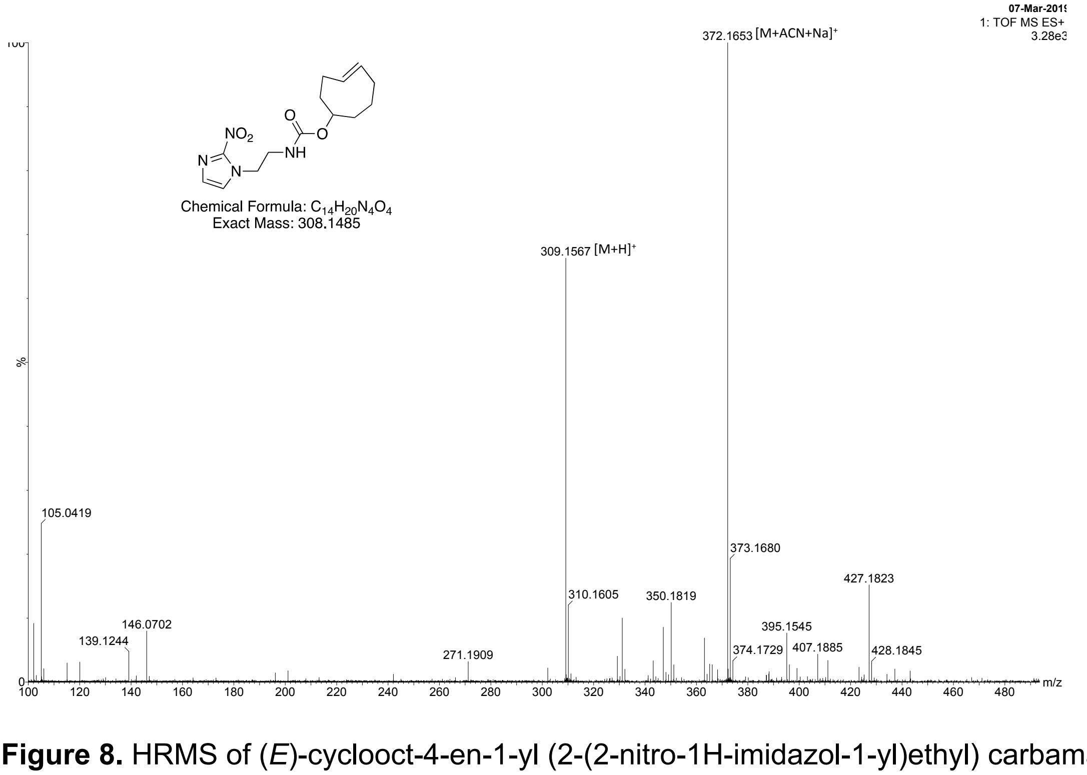


**Figure 8.** HRMS of (*E*)-cyclooct-4-en-1-yl (2-(2-nitro-1H-imidazol-1-yl)ethyl) carbamate (**8**)

#### 4.0 Toxicity Study

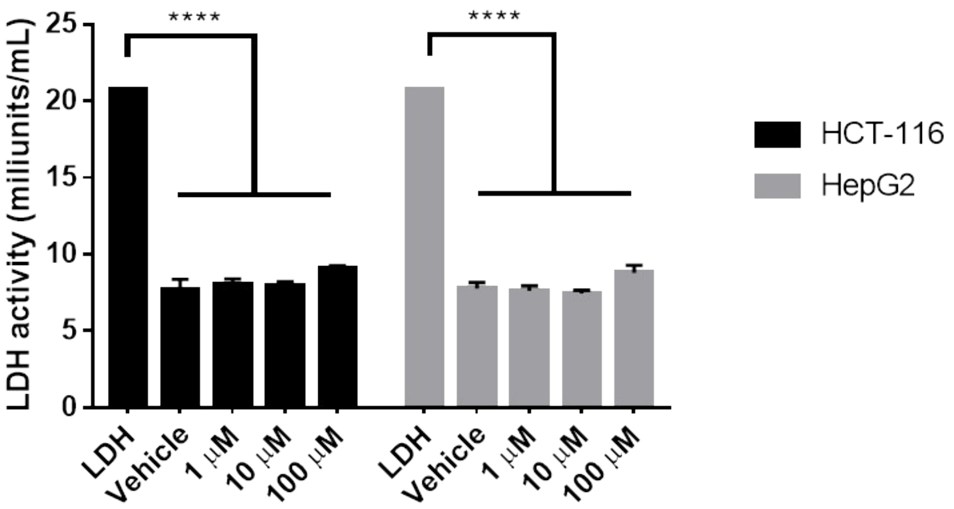


**Figure 9.** Cell membrane integrity analysis measuring LDH release to the supernatant in HCT116 and HepG2 cells upon treatment with **8** for 4 h [1]. The LDH provided by the manufacturer was utilised as positive control. The data are presented as mean ± SD, n = 6; ****$P$ < 0.0001.

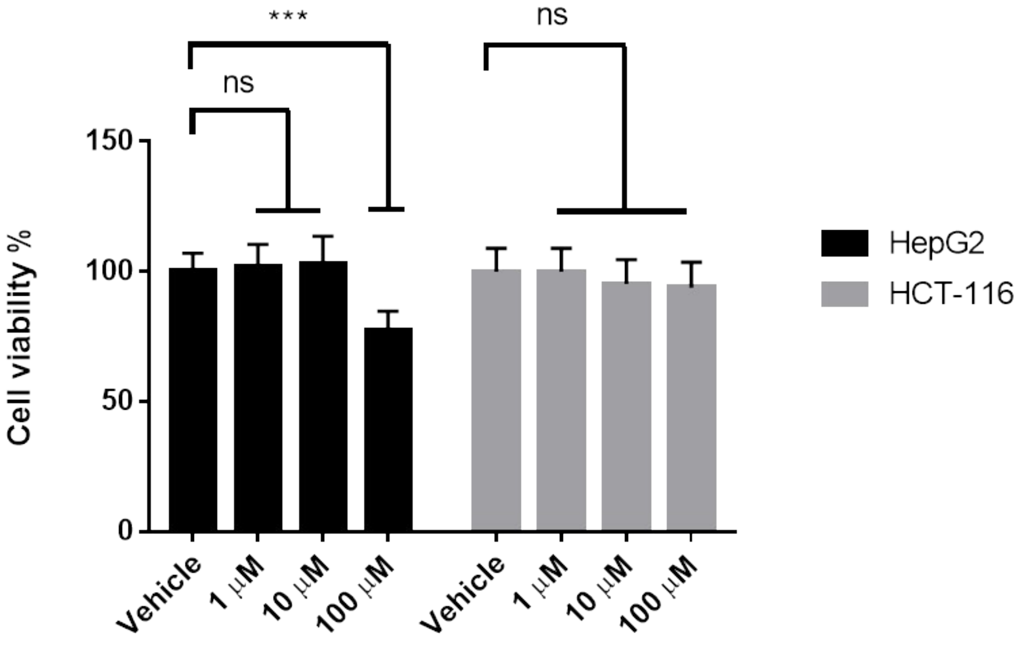


**Figure 10.** Cytotoxicity evaluation of compound **8** in HepG2 and HCT116 cells upon 72 h treatment with indicated doses of **8**. The data are presented as mean ± SD, n = 6; ***$P$ < 0.001, ns $P$ > 0.05.

## 5.0 References


1. Basuli F, Zhang X, Jagoda EM, Choyke PL, Swenson RE. Rapid synthesis of maleimide functionalized fluorine-18 labeled prosthetic group using "radio-fluorination on the Sep-Pak" method. J Label Compd Radiopharm. 2018;

2. Evans HL, Nguyen QD, Carroll LS, et al. A bioorthogonal 68Ga-labelling strategy for rapid in vivo imaging. Chem Commun. 2014;

3. Allott L, Barnes C, Brickute D, Leung SFJ, Aboagye EO. Solid-supported cyanoborohydride cartridges for automation of reductive amination radiochemistry. React Chem Eng. 2019;
